# MicroRNA 375 modulates hyperglycemia-induced enteric glial cell apoptosis and Diabetes-induced gastrointestinal dysfunction by targeting Pdk1 and repressing PI3K/Akt pathway

**DOI:** 10.1038/s41598-018-30714-0

**Published:** 2018-08-23

**Authors:** Yan Chen, Gongxiang Liu, Fuqian He, Li Zhang, Kun Yang, Huan Yu, Jinqiu Zhou, Huatian Gan

**Affiliations:** 1 The Center of Gerontology and Geriatrics, West China Hospital, Sichuan University, Chengdu, 610041 China; 2 Department of elderly digestive, Sichuan Provincial People’s Hospital, Chengdu, 610072 China; 3 Department of Gastroenterology, West China Hospital, Sichuan University, Chengdu, 610041 China

## Abstract

Diabetic neuropathy can damage systemic nervous system, including alteration of enteric nervous system and subsequent gastrointestinal dysfunction. The effect of diabetes on enteric glia cell (EGC) is not clear. We investigated the effect of diabetes and hyperglycemia on EGC, and the role of microRNA375 in modulating EGC survival *in vivo* and *in vitro*. Streptozotocin-induced diabetic mice were intraperitoneally injected with microRNA375 inhibitor or its negative control. EGC was transfected with microRNA375 inhibitor or its mimic. Diabetes mice with gastrointestinal dysfunction showed increased apoptosis of EGC (no difference in cell numbers) and gene expression of micorRNA375 in the myenteric plexus. Hyperglycemia triggered apoptosis of EGC *in vitro* with decreased expression of Pdk1 and p-Akt, but increased expression of micorRNA375. MicorRNA375 mimic induced apoptosis of EGC *in vitro* with repressed Pdk1and p-Akt. MicorRNA375 inhibitor could both prevent hyperglycemia-induced apoptosis of EGC *in vitro* and diabetes-induced gastrointestinal dysfunction *in vivo*. Our results suggest that diabetes-induced gastrointestinal dysfunction is related to increased apoptosis of EGC in the myenteric plexus. Hyperglycemia can increase the expression of microRNA375 and damage EGC survival through PI3K/Akt pathway. MicroRNA375 specific inhibition can prevent hyperglycemia induced EGC damage and diabetes-induced gastrointestinal dysfunction.

## Introduction

Diabetes mellitus is a worldwide metabolic disease and has an increasing age-standardized prevalence in adults together with population growth and ageing^1^. Gastrointestinal dysfunction is one of the most common complications in diabetic patients^2^. Some of the common complaints include vomiting, emaciation, diarrhea, constipation and fecal incontinence, and abdominal pain, all of which impair the quality of life of diabetic patients^3,4^. However, the pathogenesis of gastrointestinal dysfunction in diabetes is not well established. Previous studies reported increases of apoptosis in diabetic animal models in the dorsal root ganglion neurons, nodose ganglia^5^, and enteric nervous system^6^, which indicate diabetic neuropathy. Diabetic neuropathy is mainly considered due to hyperglycemia, dyslipidemia and metabolic syndrome^7–9^. EGC plays a critical role in enteric nervous system. In addition to modulating homeostasis of enteric neurons, involved in enteric neurotransmission and antigen presentation, emerging data demonstrate an important role of these cells in the pathophysiology of gastrointestinal motor activity^10–13^. While, the available studies regarding the chronic effects of diabetes on EGC are relatively finite and controversial^14–16^ and the effect of hyperglycemia on EGC is unknown.

MicroRNAs are endogenous small RNAs that negatively modulate gene expression in animals and plants, by targeting the untranslated region of mRNAs for cleavage or translational repression^17^. In recent years, lots of studies reported the association between microRNAs and diabetic complications^18,19^, including retina, kidney, peripheral nerves, heart and vasculature. However, the role of microRNAs in diabetes-induced gastrointestinal dysfunction is unknown. One of the major intracellular signaling pathways for neurocyte is PI3K/Akt signaling pathway. Decreased PI3K/Akt signaling has been implicated in hyperglycemia induces neuronal loss^20^ and vagal afferent neurons damage in streptozotocin-induced diabetic rats^21^. On the other hand, microRNA 375 is found to be upregulated in type 2 diabetes patients^22,23^ and could regulate PI3K/Akt signaling pathway by targeting Pdk1^24–26^.

Therefore, in our study, we hypothesized that the increased glucose concentration in diabetes may increase the expression of microRNA375, which may consequently repress the expression of Pdk1 and I3K/Akt pathway, damage EGC survival. We used high glucose treated EGC and diabetic mice to investigate the effect of hyperglycemia and diabetes on EGC, and the role of microRNA375 in modulating EGCs’ survival.

## Results

### Diabetes causes gastrointestinal dysfunction in mice

**Figure 1:**
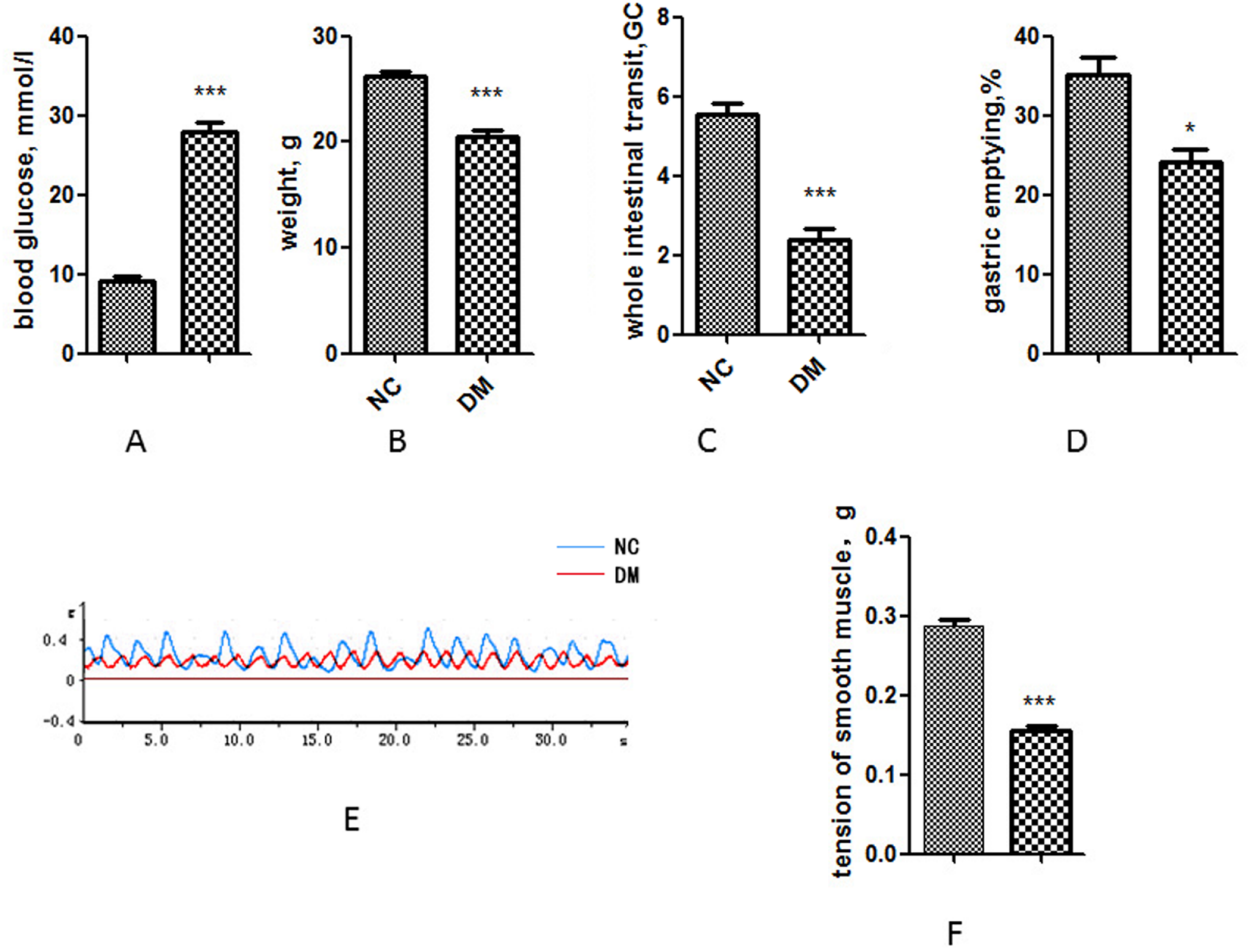
Diabetes attenuates gastric emptying, whole intestinal transit and the tension of smooth muscle. (**A**) Blood glucose of diabetic mice and normal mice 8 weeks after injection streptozotocin. (**B**) Body weight of diabetic mice and normal mice. (**C**) Whole intestinal transit estimated by GC in diabetic mice and normal mice. (**D**) Gastric emptying of diabetic mice and normal mice. (**E**) Tension of smooth muscle in diabetic mice and normal mice. NC, normal control mice; DM, diabetic mice; GC, geometric center. Each group involved 10 mice. Result expressed as mean ± SD. *P < 0.05, ***P < 0.001.

Eight weeks after injection of streptozotocin, the diabetic mice group gained significantly higher blood glucose (P < 0.001) and less weight (P < 0.001) compared with normal control group (Fig. 1A,B). Diabetic mice had significantly slower gastric emptying rate (P < 0.05) and whole intestinal transit (P < 0.001) (Fig. 1C,D). Furthermore, compared with the tension of smooth muscle of the normal group, the tension of smooth muscle of diabetic mice group was significantly reduced (P < 0.001) (Fig. 1E,F).

### Diabetes induces apoptosis of EGC in proximal colon myenteric plexus

**Figure 2:**
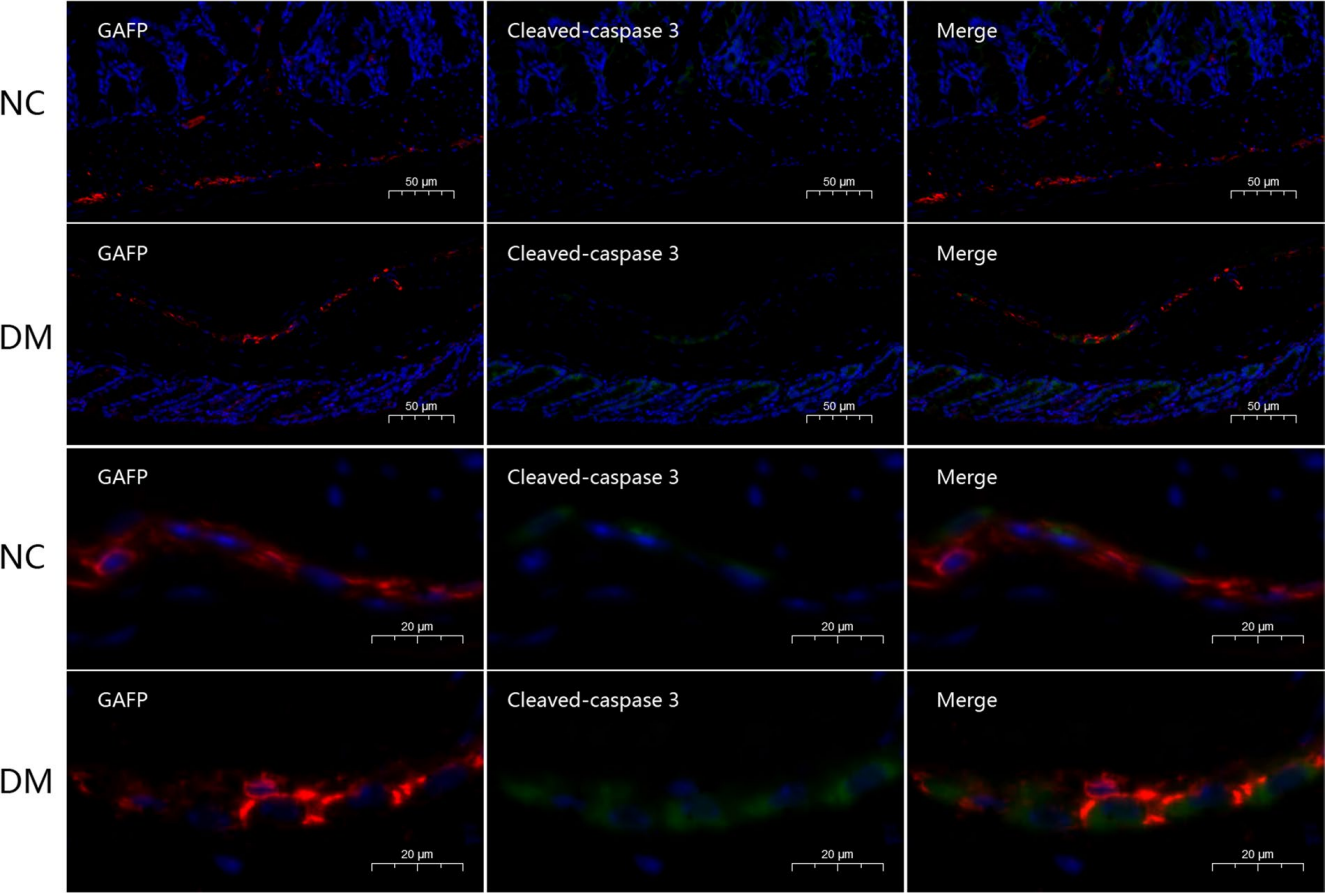
Diabetes increase apoptosis of EGC in proximal colon myenteric plexus. Whole mount double staining of proximal colon myenteric plexus for GAFP and Cleaved Caspase-3 in diabetic mice and normal control mice. NC, normal control mice; DM, diabetic mice. Each group involved 6 mice. Magnifications: the top two rows, 200x, scale bar: 50 μm; the bottom two rows, 700x. Scale bar: 20 μm.

**Figure 3:**
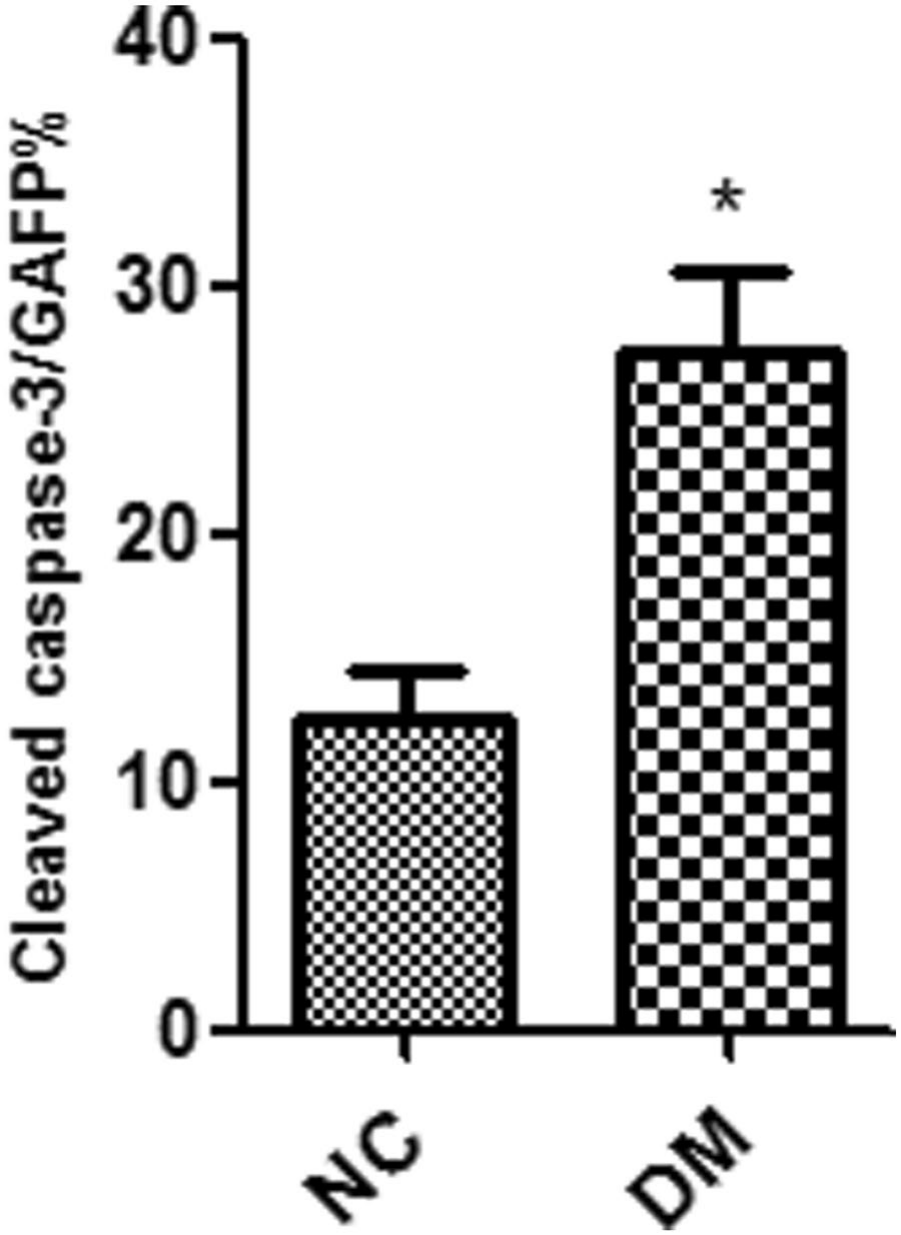
Percentage of Cleaved Caspase-3/GAFP double labeling EGCs. Whole mount stained for GAFP and Cleaved Caspase-3 in proximal colon myenteric plexus. NC, normal control; DM, diabetic mice. Result presented as mean ± SD. *P < 0.05.

To investigate the effect of diabetes on EGC, we used whole mount staining with Glial Acidic Fibrillary Protein (GAFP) and Cleaved Caspase-3 in the proximal colon myenteric plexus. The result showed a significant increase of EGC stained with both GAFP and Cleaved Caspase-3 in the myenteric plexus of the diabetic mice than that in the normal mice group (P < 0.05) (Figs 2 and 3). The number of positive GAFP cells and the expression of GAFP did not differ between the diabetic mice and the normal mice group. These results demonstrate that EGC in diabetic mice were undergoing increased apoptosis which could weaken the function of EGC.

### Hyperglycemia induces apoptosis of EGC *in vitro*

**Figure 4:**
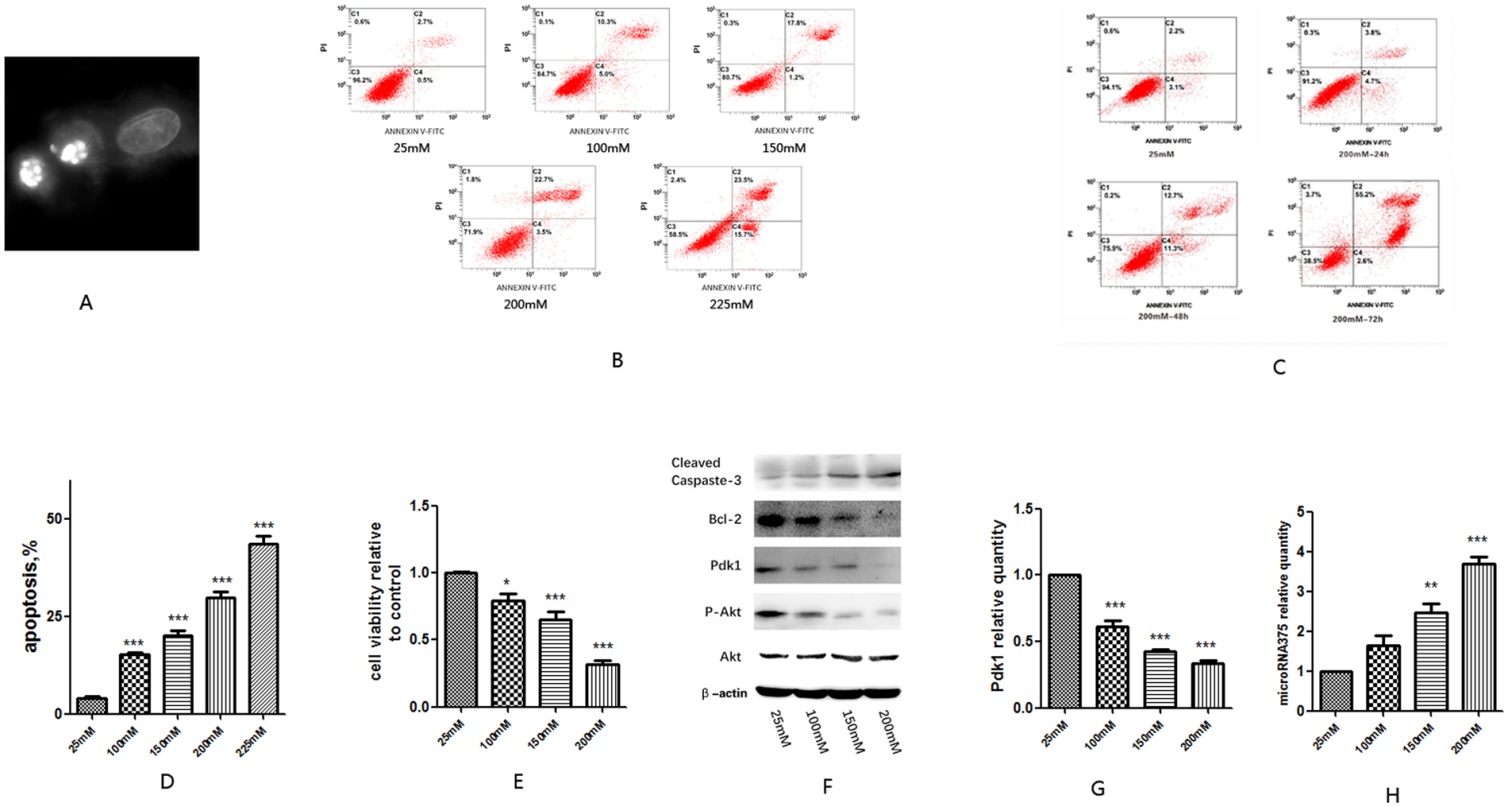
High glucose increases apoptosis of EGC *in vitro*. (**A**) DAPI staining of EGC nucleus cultured by high glucose at a concentration of 200 mmol/l for 48 hours showed apparent formation of apoptotic body and karyopyknosis. (**B**) and (**C**) Annexin V-FITC/PI flow cytometry analysis of EGC treated with different glucose concentration after 48 hours. (**D**) CCK8 evaluation of the cell viability of EGC treated with high glucose relative to control. (**E**) Western blot analysis of the Cleaved Caspase-3, Bcl-2, Pdk1 and p-Akt protein expression of EGC treated with different glucose concentration. **(F**) RT-qPCR analysis of gene expression of Pdk1in EGC treated with high glucose concentration. (**G**) RT-qPCR analysis of gene expression of microRNA375 in EGC treated with high glucose. Full-length gels and blots are included in Supplementary materials. Result presented as mean ± SD. *P < 0.05, **P < 0.01, ***P < 0.001.

To investigate the effect of hyperglycemia on EGC, we used high glucose to cultivate ECG at a concentration of 200 mM for 48 h. As we mentioned in the method part, the primary glucose level to cultivate EGC is 25 mM. Therefore, we increased the glucose level to 200 mM to simulate hyperglycemia. DAPI (4′, 6-diamidino-2-phenylindole) staining of the cell nucleus revealed apparent formation of apoptotic body and karyopyknosis (Fig. 4A). Furthermore, we used five different glucose concentrations (25, 100, 150, 200, 225 mM) to cultivate EGC for 48 h and 200 mM to treat EGC for different time points (24 h, 48 h, 72 h). Annexin V-FITC/PI flow cytometry analysis showed that the apoptosis rate of EGC accelerated from 3.93 ± 0.5 to 43.63 ± 2.13, at concentrations of 25, 100, 150, 200, 225 mM, respectively, (P < 0.001) (Fig. 4B,C). Time course-experiment showed that the apoptosis rate of EGC treated with 200 mM high glucose increased from 48 h to 96 h (Fig. 4D). CCK8 (Cell Counting Kit-8) test showed that the cell viability relative to 25 mM reduced from 0.79 ± 0.05 to 0.32 ± 0.02, at concentrations of 100, 150, 200 mM, respectively, (P < 0.05) (Fig. 4E). Western blot showed a concomitant increase of Cleaved Caspase-3 with hyperglycemia, but a converse reduction of Bcl-2 (Fig. 4F). Our results indicate that hyperglycemia can increase apoptosis of EGC *in vitro*.

### Hyperglycemia increases expression of microRNA 375 and suppresses PI3K/Akt pathway in EGC

As we mentioned before, PI3K/Akt signal pathway is essential to neurocyte survial, so we tested two key proteins of PI3K/Akt signal pathway, Pdk1 and p-Akt. Western blot showed a reduction of both Pdk1 and p-Akt in EGCs treated with hyperglycemia (Fig. 4F).

Furthermore, we tested the expression of microRNA375 (a critical upstream regulating gene of Pdk1). As we expected, the expression of microRNA375 was increased in EGCs treated with hyperglycemia (P < 0.05) (Fig. 4G,G). Our results suggest that hyperglycemia can increase the expression of microRNA375 and repress PI3K/Akt signal pathway.

### Transfection of microRNA375 inhibitor prevents high glucose-induced apoptosis of EGC ***in vitro***

**Figure 5:**
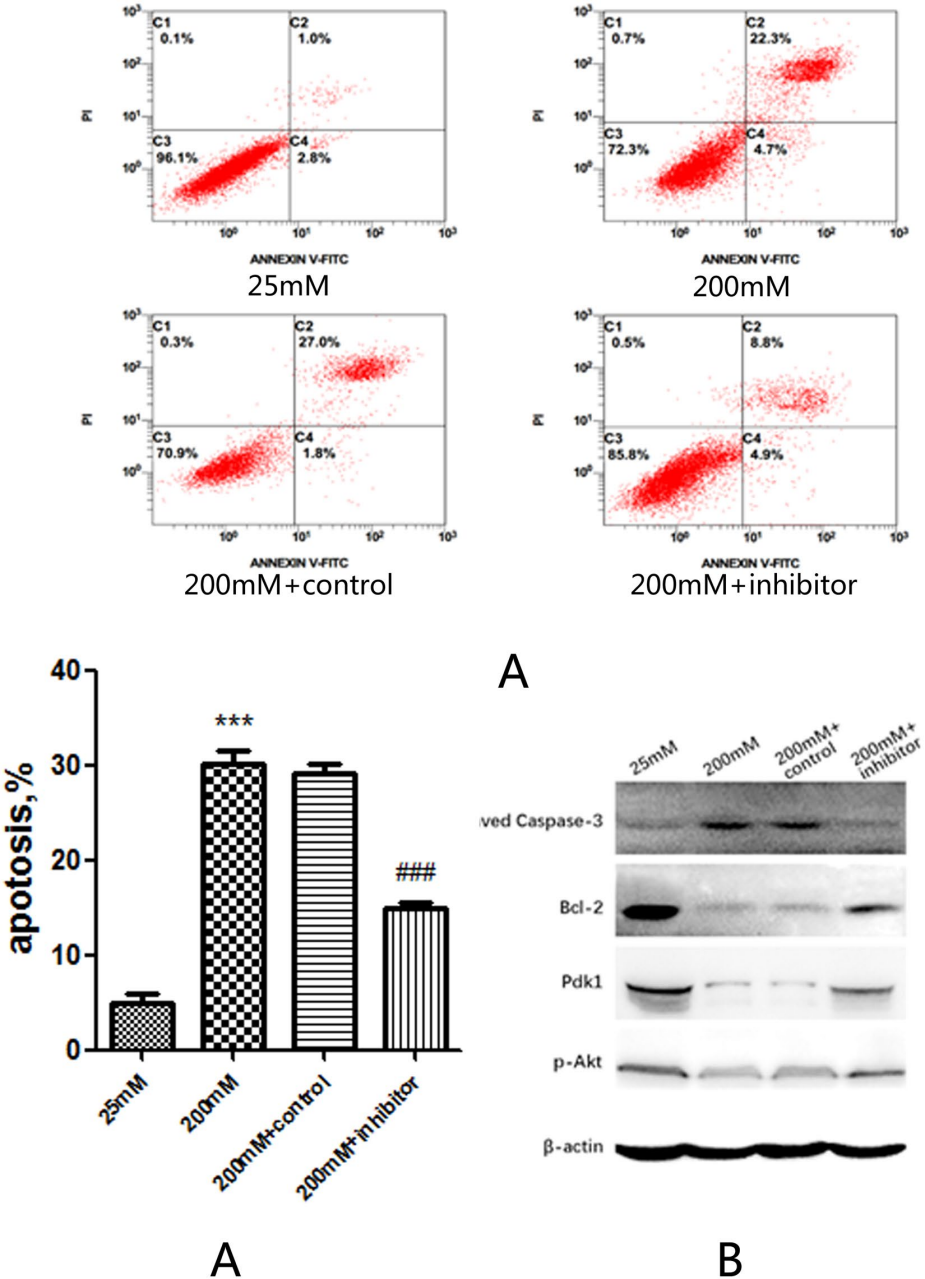
Transfection of microRNA375 inhibitor *in vitro*. (**A**) AnnexinV- FITC/PI flow cytometry analysis of apoptosis of EGC transfected with microRNA375 inhibitor or microRNA375 inhibitor negative control. (**B**) Western blot analysis of Cleaved Caspase-3, Bcl-2, Pdk1 and p-Akt in EGC transfected with microRNA375 inhibitor or miRNA375 inhibitor negative control. 200 mM + control, EGC treated with 200 mM glucose concentration and transfected with microRNA375 negative control. 200 mM+ inhibitor, EGC treated with 200 mM glucose concentration and transfected with microRNA375 inhibitor. Full-length gels and blots are included in Supplementary materials. Result presented as mean ± SD. ***P < 0.001, apoptosis of EGC treated with 200 mM glucose compared to EGC treated with 25 mM glucose; ^###^P < 0.001, apoptosis of EGC treated with 200 mM glucose + microRNA375 inhibitor compared to EGC treated with 200 mM glucose.

To further investigate the role of microRNA375 in EGC and whether the increase of microRNA375 could repress PI3K/Akt signal pathway through targeting Pdk1, we transfected EGC with a microRNA375 specific inhibitor or its negative control followed by high glucose treatment (200 mM) for 48 hours. Annexin V-FITC/PI flow cytometry analysis showed that high glucose-induced apoptosis of EGCs was significantly decreased when microRNA375 was inhibited compared with microRNA375 inhibitor negative control (P < 0.001) (Fig. 5A). Western blot analysis also showed a significant reduction of Cleaved Caspase-3 and increase of Bcl-2 in EGC transfected with microRNA375 inhibitor (Fig. 5B). Western blot analysis further revealed an increase of Pdk1 and p-Akt in high glucose cultivated EGC transfected with microRNA375 inhibitor (Fig. 5B). These results show that inhibition of microRNA375 can partly abrogate hyperglycemia-induced apoptosis of EGC through increasing the expression of Pdk1 and activating PI3K/Akt pathway.

### Transfection of microRNA375 mimic induces apoptosis of EGC *in vitro*

**Figure 6:**
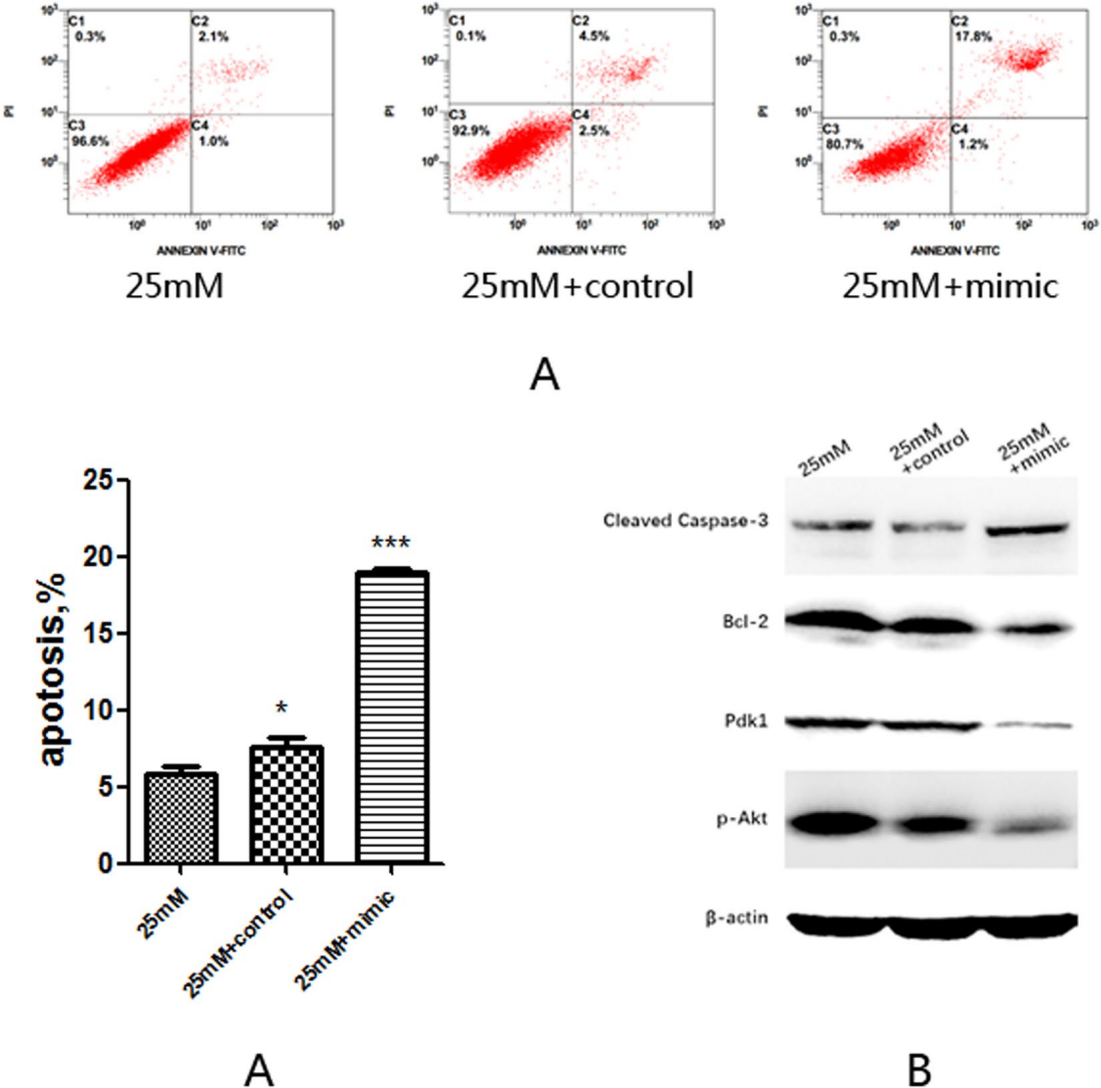
Transfection of microRNA375mimic *in vitro*. (**A**) AnnexinV- FITC/PI flow cytometry analysis of apoptosis of EGC transfected with microRNA375mimic or microRNA375 mimic negative control. (**B**) Western blot analysis of Cleaved Caspase-3, Bcl-2, Pdk1 and p-Akt in EGC transfected with microRNA375 mimic or miRNA375 mimic negative control. 25 mM + control, EGC treated with 25 mM glucose concentration and transfected with microRNA375 mimic negative control. 25 mM + mimic, EGC treated with 25 mM glucose concentration and transfected with microRNA375mimic. Full-length gels and blots are included in Supplementary materials. Result presented as mean ± SD. *P < 0.05, ***P < 0.001.

To further confirm the role of microRNA375 in modulating EGC survival, EGCs cultured with normal glucose concentration were transfected with microRNA375mimic or its negative control for 48 hours. Annexin V-FITC/PI flow cytometry analysis showed a significant increased apoptosis of EGC transfected with microRNA375 mimic compared with its mimic negative control (P < 0.05) (Fig. 6A). Western blot analysis showed a significant increase of Cleaved Caspase-3 and reduction of Bcl-2 in EGC transfected with microRNA375mimic (Fig. 6B). Western blot analysis also showed a significant decrease of Pdk1 and p-Akt in EGC transfected with microRNA375 mimic (Fig. 6B). Our results demonstrate that microRNA375 can modulate EGC survival through targeting Pdk1 and inhibiting PI3K/Akt pathway.

### Diabetic mice increase expression of microRNA 375 in proximal colon myenteric plexus

**Figure 7:**
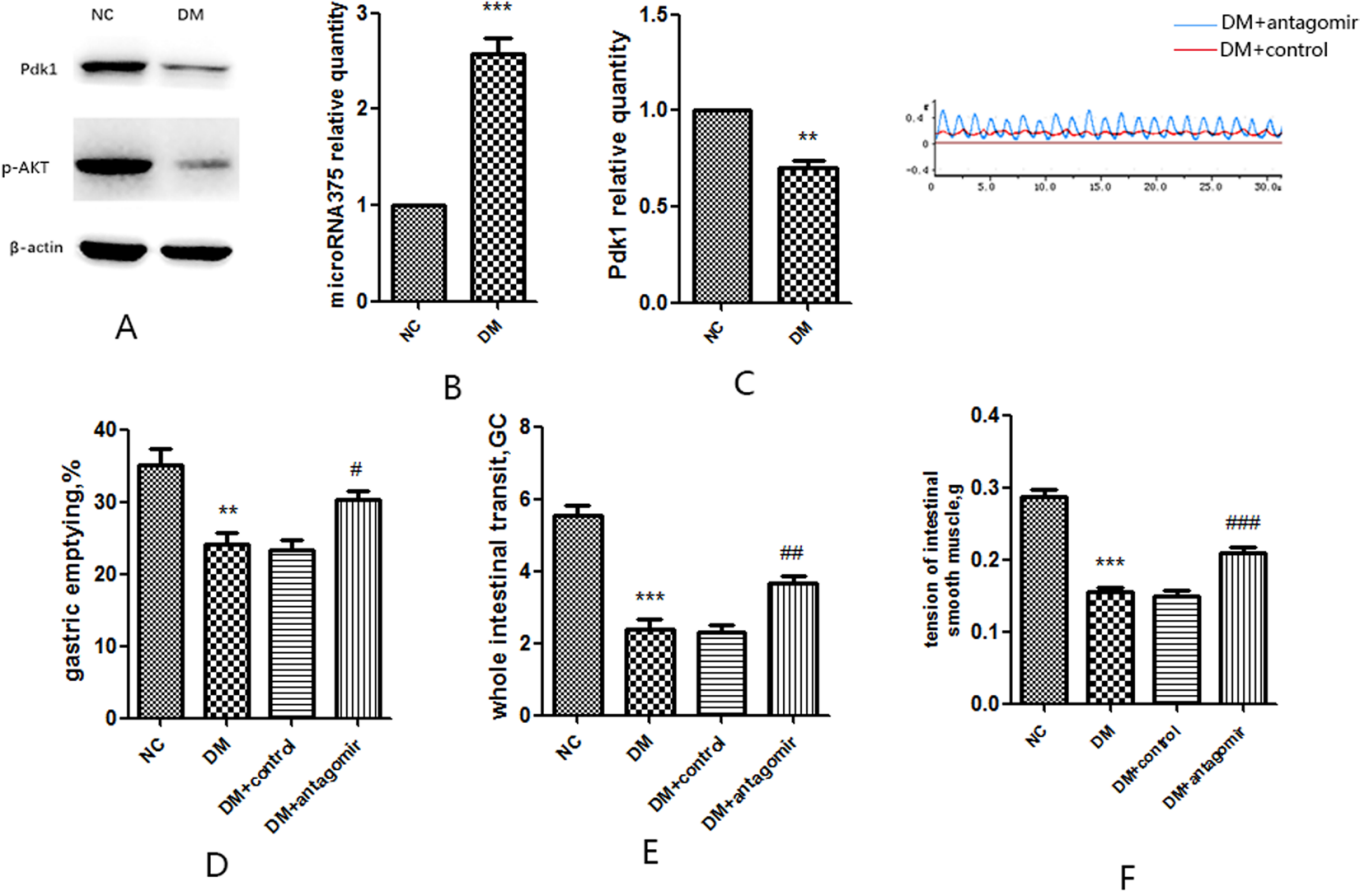
Systemic injection of microRNA375antagomir *in vivo*. (**A**) Western blot analysis of Pdk1 and p-Akt in diabetic mice and normal control mice. (**B**) RT-qPCR analysis of microRNA 375expression in diabetic mice and normal control mice. (**C**) Gene expression of Pdk1 in diabetic mice and normal control mice. (**D**) Intraperitoneal injection of microRNA375antagomir prevented delayed gastric emptying in diabetic mice. (**E**) Inhibition of microRNA375antagomir ameliorates delayed whole intestinal transit in diabetic mice. (**F**) Transfection of microRNA375antagomir increases the tension of smooth muscle in diabetic mice. NC, normal control mice; DM, diabetic mice; DM + control, diabetic mice transfected with microRNA375antagomir negative control; DM+ antagomir, diabetic mice transfected with microRNA375antagomir. Each group involved 10 mice. Result presented as mean ± SD. diabetic mice compared to normal control mice: *P < 0.05, **P < 0.01, ***P < 0.001; diabetic mice treated with microRNA375antagomir compared to diabetic mice: ^#^P < 0.05, ^##^P < 0.01, ^###^P < 0.001.

To further investigate the roll of microRNA375 *in vivo*, we tested the gene expression microRNA 375 and Pdk1 in proximal colon myenteric plexus in both diabetic mice and normal mice. Consistent with the previous results, we found that both Pdk1 and p-Akt protein were lessoned in the proximal colon myenteric plexus of diabetic mice compared with that in normal control mice group (Fig. 7A). Importantly, the gene expression of microRNA 375 was higher in diabetic mice than normal control mice group (P < 0.01) (Fig. 7B). On the contrary, the gene expression of Pdk1 was lower in diabetic mice (P < 0.01) (Fig. 7C).

### Systemic injection of microRNA375antagomir prevents gastrointestinal dysfunction in mice

Finally, to further confirm the role of microRNA375 in diabetic mice, we used systemic injection of microRNA375antagomir or its negative control in diabetic mice for four weeks. As a result, inhibition of microRNA375 prevented delayed gastric emptying (P < 0.05) and whole intestinal transit (P < 0.01) in diabetic mice (Fig. 7D,E). Further, the tension of smooth muscle of diabetic mice was also increased in mice treated with microRNA375 antagomir compared to the negative control mice (P < 0.001) (Fig. 7F).

## Discussion

In the present study, we used a culture system of EGC *in vitro* and a streptozotocin -induced diabetic mice model *in vivo*. We demonstrated that diabetes-induced gastrointestinal dysfunction is associated with an increase of apoptosis of EGC in myenteric plexus, but not the number of EGC. Next, we found that hyperglycemia triggers apoptosis of EGC *in vitro*, attenuates their cell viability, represses Pdk1 and PI3K/Akt pathway, and increases expression of micorRNA375. Furthermore, we demonstrated that inhibition of micorRNA375 can ameliorate hyperglycemia-induced EGC apoptosis. On the contrary, mimic the effect of micorRNA375 can increase the apoptosis of EGC. Finally, systemic inhibition of micorRNA375 can alleviate diabetes-induced gastrointestinal dysfunction *in vivo*.

We used mice with streptozotocin-induced diabetes to study the effect of diabetes on gastrointestinal motility. In agreement with previous studies^22,27^, we also found delayed gastric emptying rate, slower intestinal transit, and declined tension of intestinal smooth muscle in diabetic mice. Chloe Stenkamp^16^ and his colleague reported that obesity and diabetes progressed in high-fat diet mice did not alter S100β, Sox10 and GFAP expression in myenteric of EGC. Sox10 showed a diet independent, age-associated decline in EGC. So we used Cleaved-caspase 3/GAFP double immunofluorescence labeling to observe EGC in proximal colon myenteric plexus. We found no difference of the expression of GAFP and the number of GAFP positive cells in myenteric plexus between the diabetic mice and the normal mice. However, we found that the Cleaved-caspase 3/GAFP double labeling cells in the diabetic mice were significantly more than that in the normal mice group.

To study weather hyperglycemia in diabetes can induce apoptosis of EGC we treated EGCs with high glucose concentration *in vitro*. We found that as we increase glucose concentration to culture EGC, the apoptosis rates of EGC also increased. After then we tried to investigate the possible mechanism involved in hyperglycemia-induced EGC apoptosis. It is reported that apoptotic death in hyperglycemia is related to hyperglycemia-induced oxidative stress, inhibited PI3K/Akt and ERK1/2 MAPK signaling pathway^28^, depolarization of neurons and increased intracellular calcium^29^, mitochondrial membrane depolarization, cleavage of caspases^30^. In our study, we observed a significant increase of Cleaved-caspase 3 and decrease of Pdk1 and p-Akt protein, which indicate an inhibition of PI3K/Akt pathway in high glucose cultured EGC. However, the upstream gene regulation is still unknown. Thus, we tried to find out the possible involved gene regulation mechanism.

MicroRNAs, a non-coding RNAs, are reporeted to regulate gene expression by translational repression^17^ and have successful translational application^31^. As we mentioned before, microRNA375 was reported to be upregulated in diabetes patients, so we next tested the expression of microRNA375 in the myenteric plexus of diabetic mice and hyperglycemia treated EGC. We found that the expression of microRNA375 were both up-regulated in hyperglycemia treated EGC and the myenteric plexus of diabetic mice. Furthermore, by using mimic to up-regulate and inhibitor to down-regulate microRNA375 in EGC, we demonstrated that up-regulated expression of microRNA375 can induce EGC apoptosis, down-regulated expression of microRNA375 can prevent the hyperglycemia-induced EGC apoptosis, and these effects of microRNA375 were partly through targeting Pdk1 and suppressing PI3K/Akt pathway. Finally, through systemic inhibition of microRNA375 to diabetes mice, we found that inhibition of microRNA375 could partly ameliorate the diabetes-induced gastrointestinal dysfunction.

Our results contribute to a better understand of the molecular mechanism of diabetes-induced gastrointestinal dysfunction. It is proved that EGC are critical for gastrointestinal function, elimination of EGC may alter gastrointestinal function^32^, and transplantation of neural stem cells may improve gastrointestinal function in diabetic mice through supplementation of GAFP-positive cells^33^. Previous study has also reported that glial cell line–derived neurotrophic factor (GDNF), which is critical for the survival of enteric neurons, could rescue hyperglycemia-induced diabetic enteric neuropathy^20^. This may also explain how hyperglycemia-induced EGC apoptosis alters the gastrointestinal motility because increase of apoptosis of EGC may damage the function of EGC and reduce the production of GDNF. Further studies need to investigate the influence of diabetes and hyperglycemia on the function of EGC. Most importantly, some studies reported that microRNA were up-regulated in several cancers^25,31,34^, future studies are also needed to access the role of microRNA in cancer development and microRNA-specific therapy.

In conclusion, our results suggest that diabetes-induced gastrointestinal dysfunction is related to the increased hyperglycemia-induced apoptosis of EGC in ENS. Hyperglycemia triggers the apoptosis of EGC through up-regulation of microRNA375 and repression of PI3K/Akt pathway. MicroRNA375 modulates EGC survival by targeting Pdk1 and repressing PI3K/Akt pathway. Mimic the effect of microRNA375 could increase EGC apoptosis. Inhibition of microRNA375 could prevent the damage effect of hyperglycemia on EGC and diabetes-induced gastrointestinal dysfunction. Our results suggest a novel insight of the mechanism of diabetes-induced gastrointestinal dysfunction and provide a therapeutic potential of micro-RNA specific method in human gastrointestinal diseases.

## Materials and Methods

### Preparation of experimental animals

All animals’ procedures were approved by the Animal Ethics Committee of West China Hospital, Sichuan University. All methods were performed in accordance with the relevant guidelines and regulations. C57BL/6 male mice, about eight weeks old, weighing 18–22 g, were obtained from the Experimental Animal Center of Sichuan University. All mice were tested with a random blood glucose measured by Glucose Analyzer (Roche, Shanghai, China) as baseline blood glucose concentration and then randomly assigned to normal mice group or diabetes mice group. Each group had 10 mice. All mice were fasted overnight, and injected intraperitoneally with 150 mg kg^−1^ streptozocin (Sigma, St. Louis, MO, USA) dissolved in citrate buffer (pH 4.5, Sigma, St. Louis, MO,USA) or with an equal volume of citrate buffer. 72 h later, mice with a random blood glucose higher than16.7 mM were defined as diabetic mice^35–37^. Then mice were feed 8 weeks after injection. Glucose levels were monitored weekly and mice did not meet this criterion were excluded.

### EGC culture

EGCs were obtained from ATCC and cultured in DMEM medium (Invitrogen, Carlsbad, CA, USA) supplemented with 10% (v/v) heat-inactivated fetal bovine serum (Gibco, Invitrogen). The original glucose concentration of DMEM medium to cultivate EGC is 25 mM (mmol L^−1^). We used D-(+)-Glucose (Sigma, St. Louis, MO, USA) to modulate different high glucose concentrations (100, 150, 200, 225 mM).

### Transfection of microRNA375 inhibitor ***in vitro***

EGCs were transfected with a chemically modified antisense oligonucleotide- microRNA375 inhibitor (RiboBio, Guangzhou, China) at a concentration of150nmol/L. MicroRNA375 inhibitor negative control (oligonucleotide mismatching microRNA375) was transfected as negative control. All of our transfections used transfection reagent (RiboBio, Guangzhou, China) following the manufacturer’s instructions. Apoptosis was measured by flow cytometer and western blot 48 hours later. The sequences of microRNA375 inhibitor and microRNA375 inhibitor negative control are described in Supplementary materials.

### Transfection of microRNA375 mimic ***in vitro***

EGCs were transfected with a chemically simulative oligonucleotide-microRNA375 mimic and its negative control (RiboBio), respectively, at a concentration of100nmol/L. All transfections used transfection reagent from RiboBio following the manufacturer’s instructions. Apoptosis was measured by flow cytometer and western blot 48 hours later. The sequences of microRNA375 mimic and microRNA375 mimic negative control are described in Supplementary materials.

### Systemic injection of microRNA375antagomir in mice

Diabetic mice were randomized into treatment and negative control groups. Each group had 6 mice. The treatment group was injected intraperitoneally with chemically synthesized microRNA375antagomir (antisense oligonucleotide). The negative control group was injected intraperitoneally with synthesized microRNA375antagomir negative control (oligonucleotide mismatching microRNA375). Mice were injected with a daily dose of 8 mg kg^−1^ for three consecutive days, and then a weekly maintenance injection for three weeks. The sequences of microRNA375antagomir and microRNA375antagomir negative control are described in Supplementary materials.

Measurement of whole intestinal transit and gastric emptying, tension of smooth muscle, EGC apoptosis rate, Cell Viability, GFAP/Cleaved Caspase-3 double labeling, DAPI staining, quantitative- real time PCR are described in Supplementary materials.

### Statistical analysis

Data are presented as the means ± standard deviation (SD). One-way analysis of variance (one-way ANOVA) or the 2-tailed Student’s *t*-test was performed for inter-group comparisons. Nenman-keuls was used for inter-group comparisons in more than two groups. P values of less than 0.05 were considered to be statistically significant. SPSS 17.0 was used for statistical analysis.

## Electronic supplementary material

Supplementary materials
