# Supplementary material for: MicroRNA 375 modulates hyperglycemia-induced enteric glial cell apoptosis and Diabetes-induced gastrointestinal dysfunction by targeting Pdk1 and repressing PI3K/Akt pathway
Source: Sci Rep. 2018 Aug 23;8:12681. doi: 10.1038/s41598-018-30714-0 (PMC6107553; doi:10.1038/s41598-018-30714-0)
Supplement: Supplementary file 1 — Supplementary materials [file 41598_2018_30714_MOESM1_ESM.docx]

MicroRNA 375 modulates hyperglycemia-induced enteric glial cell apoptosis and Diabetes-induced gastrointestinal dysfunction by targeting Pdk1 and repressing PI3K/Akt pathway

Yan Chen^1^, Gongxiang Liu^1^, Fuqian He^1^, Li Zhang^2^, Kun Yang^1^, Huan Yu^3^, Jinqiu Zhou^1^, Huatian Gan^1, 3*^

**Sequences of transfected genes**

| Gene | | sequence | | |
| --- | --- | --- | --- | --- |
| micrOFF ^TM^ rno-miR-375-3p inhibitor | | | UCACGCGAGCCGAACGAACAAA | |
| micrOFF TM inhibitor Negative Control | | CAGUACUUUUGUGUAGUACAAA | | |
| micrON ^TM^ rno-miR-375-3p mimic | Forward UUUGUUCGUUCGGCUCGCGUGA | | | |
|  | | Reverse UCACGCGAGCCGAACGAACAAA | | |
| micrON ^TM^ mimic Negative Control | | Forward UUUGUACUACACAAAAGUACUG  Reverse CAGUACUUUUGUGUAGUACAAA | | |
| micrOFF ^TM^ rno-miR-375-3Antagomir | | UCACGCGAGCCGAACGAACAAA | | |
| micrOFF ^TM^ Antagomir Ncontrol | | | | CAGUACUUUUGUGUAGUACAAA |

**Methods**

**Measurement of intestinal transit and gastric emptying**

Intestinal transit and gastric emptying were measured as previously described ([1](#_ENREF_1)). Briefly, after an overnight fast, mice received 0.1 mL of phenol red solution (0.5 mg mL^-1^) mixed with 1.5% methylcellulose by gavage. Thirty minutes later, the mice were sacrificed by cervical dislocation. The entire stomach was carefully isolated, ligated just above the cardia and below the pylorus. The entire colon was divided into nine segments of equal length. The amount of phenol red was measured spectrophotometrically (Beckman Instruments Inc., Palo Alto, CA, USA) at 560 nm. The percentage of gastric emptying was defined as the ratio between the amount of phenol red recovered from the stomach and the amount of phenol red ingested into the stomach and calculated as the following formula: [(A560 reference- A560 sample)/A560 reference] × 100.

Intestinal transit was assessed using a parameter called geometric center (GC) and calculated as the following formula: Geometric center=Σ (counts of phenol red per segment × A segment number)([2](#_ENREF_2)).

**Measurement of tension of intestinal smooth muscle in mice duodenum**

Tension of intestinal smooth muscle was measured by HW200S/HW201S experiment system following the manufacturer's instructions. First, we killed mice by cervical dislocation and carefully isolated the entire stomach and the intestinal. Below the pylorus was duodenum. 1.5-2cm duodenum muscle strips were obtained from living mice. Then, the strips were mounted between 2 hooks which connected to Tension transducer by surgical lines and placed between 2 platinum electrodes in 50 ml chambers. The chambers contained Krebs buffer at 37°C and continuously gassed with 95% O2 and 5% CO2. After the measurement system was set up, we first adjusted the baseline tension to 0.1g-0.2g through adjusting the height of Tension transducer and the tension of the line which connected the strip and the Tension transducer. Ten minutes after the curve got stable, we recorded the data for each samples. Tension was monitored by BL-420F biological signal collecting and processing system. Tension of intestinal smooth muscle was calculated as the following formula: F = m × g. For the figures which illustrated the tension of intestinal smooth muscle were automatically generated, we did not change the units on the figures.

**GFAP/ Cleaved Caspase-3 double labeling in mice proximal colon myenteric plexus**

Protocol for whole mount, fixation and histochemical staining was based on previously described([3](#_ENREF_3)). Antibodies for double immunofluorescence staining were performed using GFAP (mouse, 1:100, Pharmingen, CA, USA) and Cleaved Caspase-3(rabbit, 1:200, Cell Signaling, MA, USA). The results were measured by visual count under a 200× magnification in the myenteric plexus. We first count the total number of GFAP positive cells and then counted the number of Cleaved Caspase-3 positive cells among these cells. At least 6 sections were scored for each slides. Each group involved 6 mice. The proportion of GFAP positive/Caspase 3 positive cells was calculated as the following formula: number of GAFP and Cleaved Caspase-3 double positive cells/ GAFP positive cells.

**Longitudinal muscle myenteric plexus preparations and western blot analysis**

Isolation of longitudinal muscle myenteric plexus was performed as previous described([4](#_ENREF_4)). Total protein extracts from mice and cells were prepared as previous described ([5](#_ENREF_5)). The antibodies and dilutions were as follows: GFAP (mouse, 1:500, Pharmingen), cleaved-Caspase-3(rabbit, 1:1000, Cell Signaling), bcl-2 (rabbit, 1:1000,absin, Shanghai, China), Pdk1 (rabbit, 1:800, Cell Signaling), p-Akt (rabbit, 1:1000, Cell Signaling), β-Actin (rabbit, 1:1000, Cell Signaling) , horseradish peroxidase conjugated anti-mouse and anti-rabbit IgG secondary antibodies (1:2000, Cell Signaling Technologies). A semi quantitative measurement of band density was measured by a fluorchem imaging system (Alpha Innotech Corp, San Leandro, CA, USA).

**DAPI staining**

We used 4', 6-diamidino-2-phenylindole staining (DAPI Staining Solution, Beyotime, Beijing, China) to observe cell’ nucleus according to the manufacturer's instructions by fluorescence microscopy (Olympus, Tokyo, Japan).

**Measurement of apoptosis rate**

Cell apoptosis rate was measured by Annexin V and Propidium Iodide (Annexin V-FITC/PI Apoptosis Detection Kit, 4A Biotech Co., Ltd, Beijing, China) according to the manufacturer's instructions. Briefly, Annexin V and PI were added to EGC cells prepared and incubated in the dark for 15 minutes at room temperature followed by measurement of apoptosis by FACSCalibur flow cytometer (BD Biosciences). Results were measured by visual count under a 200× magnification in the myenteric plexus. We first count the total number of GFAP positive cells and then counted the number of Cleaved Caspase-3 positive cells among these cells. At least 6 sections were scored for each slides. Each group involved 6 mice. The proportion of GFAP positive/Caspase 3 positive cells was calculated as the following formula: number of GAFP and Cleaved Caspase-3 double positive cells/ GAFP positive cells.

**Measurement of Cell Viability**

Cell viability was measured by tetrazolium salt WST-8 [2-(2-methoxy-4-nitrophenyl)-3-(4-nitrophenyl)-5- (2, 4-disulfophenyl)-2H-tetrazolium, monosodium salt] (Cell Counting Kit-8, CCK-8, Dojindo Laboratories, Kumamoto, Japan) according to the manufacturer's instructions. CCK-8 solution was added to the cells and incubated for 4 hours and the absorbance of each well at 450 nm was measured by an ultraviolet spectrophotometer.

**Quantitative- real time PCR**

Total RNA was extracted using Eastep® Super Total RNA Extraction Kit (Promega, Beijing, China) according to the manufacturer's protocol. The complementary DNA was made using First strand cDNA synthesis kit (Thermo Scientific, DE, USA). The reverse transcription polymerase chain reaction (RT-PCR) was performed using 2*SYBR green qPCR master mix (Thermo Scientific, DE, USA). The following primers were used:

Mir375-5’-UUUGUUCGUUCGGCUCGCGUGA-3’,

**Pdk1** FW-5’ GTGCCCATTCAGTCCAGTGT -3’

REV-5’- AAGGGGTTGGTGCTTGGTC -3’

**GAPDH** FW-5’ GGGCTTGTCTCTGGTGTGAC -3’

REV-5’- GTTGCTGTTGAAGTCGCAGG -3’

**Reference**

1. Izbeki F, Wittmann T, Jancso G, et al. Inhibition of gastric emptying and small intestinal transit by ethanol is mediated by capsaicin-sensitive afferent nerves. Naunyn Schmiedebergs Arch Pharmacol 2002;365:17-21.

2. Qiu WC, Wang Zg Fau - Lv R, Lv R Fau - Wang W-G, et al. Ghrelin improves delayed gastrointestinal transit in alloxan-induced diabetic mice.

3. Bassotti G. The role of glial cells and apoptosis of enteric neurones in the neuropathology of intractable slow transit constipation. Gut 2006;55:41-46.

4. Stenkamp-Strahm C, Patterson S, Boren J, et al. High-fat diet and age-dependent effects on enteric glial cell populations of mouse small intestine. Autonomic Neuroscience 2013;177:199-210.

5. Anitha M, Gondha C, Sutliff R, et al. GDNF rescues hyperglycemia-induced diabetic enteric neuropathy through activation of the PI3K/Akt pathway. J Clin Invest 2006;116:344-56.

**Full-length gels and blots**


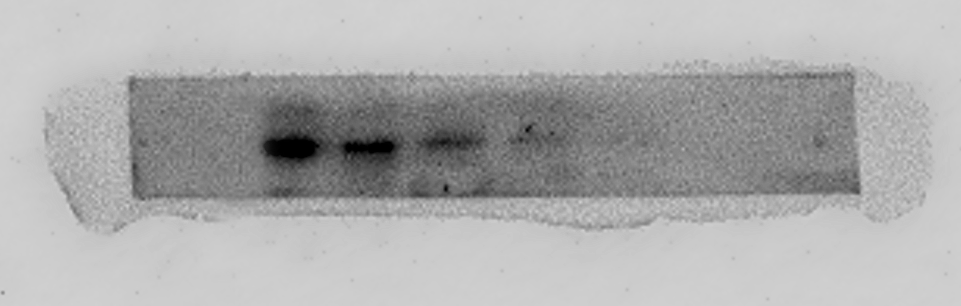


FIGURE 4. F bcl-2





FIGURE 4. F Pdk1





FIGURE 4. p-Akt





FIGURE 4. F Akt


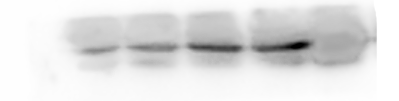


FIGURE 4. F Cleaved Caspase-3





FIGURE 4. F β-actin





FIGURE 5.B Pdk1


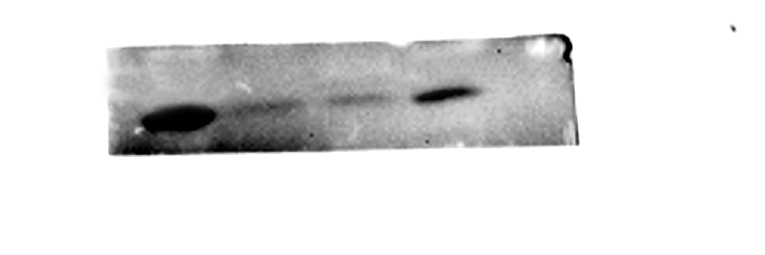


FIGURE 5.B bcl-2





FIGURE 5.B p-Akt





FIGURE 6.B Cleaved Caspase-3





FIGURE 6.B bcl-2
